# Nanoparticles as tools for enhancing plant resistance to biotic stress in the context of climate change

**DOI:** 10.1111/ppl.70227

**Published:** 2025-04-16

**Authors:** Kettlin Ruffatto, Luana Vanessa Peretti Minello, Bárbara Gottardi Furtado, Liana Johann, Raul Antonio Sperotto

**Affiliations:** ^1^ Graduate Program in Biotechnology University of Vale do Taquari – Univates Lajeado RS Brazil; ^2^ Life Sciences Area University of Vale do Taquari – Univates Lajeado RS Brazil; ^3^ Graduate Program in Plant Physiology Federal University of Pelotas Pelotas RS Brazil

## Abstract

In the face of climate change, agriculture is increasingly challenged by shifting dynamics of biotic stresses, including the intensified spread of pests and pathogens. Traditional control methods, often reliant on chemical pesticides, are associated with environmental degradation and potential health risks. Nanoparticles (NPs) present a promising, sustainable alternative for enhancing plant resistance to biotic stresses, potentially revolutionizing agricultural practices. This mini‐review explores the mechanisms through which NP‐based formulations (such as metal‐based NPs, chitosan, and silica) induce plant responses and bolster defences against pathogens and pests. By enhancing plant resilience without the environmental downsides of conventional pesticides, NPs could support a more sustainable approach to crop protection. This review also highlights the potential risks in expanding the use of NPs in agriculture, urging more studies to explore these technologies as a sustainable approach to managing crops in a changing climate.

## 1 INTRODUCTION

As global agricultural systems face unprecedented challenges from climate change, the demand for sustainable solutions to enhance crop resilience has never been more urgent (Verma et al., 2024a). Climate change exacerbates biotic stressors such as fungi, bacteria, insects, and mites, severely threatening global food security (Shree et al., 2022). Rising temperatures, increased CO₂ concentrations, altered precipitation patterns, and extreme weather events contribute to the spread of pests and pathogens, creating more favourable conditions for infestations and reducing plant immunity (Yactayo‐Chang and Block, 2023). The control of phytopathogens is primarily carried out using chemical macromolecules, which have limited success and directly impact the beneficial microbiota in the soil, as well as animal and human health, in addition to promoting pest resistance (Rajpal et al., 2024).

In this context, the use of nanoparticles (NPs) in agriculture offers a cutting‐edge approach to enhance plant resistance to biotic stress (Zhang et al., 2024). This technology has shown promise in reducing dependency on chemical pesticides while potentially improving crop yield and resilience (Shukla et al., 2024). Due to their reduced size (1‐100 nm) and high reactivity, NPs can promote complex interactions with various organic and inorganic molecules (Otari et al., 2024). Additionally, other characteristics such as adjustable surface properties, porosity, adaptable morphology, and a higher surface: volume ratio, facilitate access to plant cells, allowing for controlled and targeted delivery of nutrients, hormones, and defence agents directly to target tissues (Saritha et al., 2022; Francis et al., 2024a). Therefore, they can influence metabolic pathways related to natural plant defence and protection, increasing enzymatic antioxidant activity, the production of specialized metabolites, and the regulation of defence hormones, helping plants respond more quickly and efficiently to recurring stress and inducing systemic resistance against pathogen and herbivore attacks (Otari et al., 2024; Francis et al., 2024b).

Various NPs possess antimicrobial properties, particularly those derived from metal ions such as Ag and Zn, which can directly control fungi, bacteria, and other phytopathogenic pests (Li et al., 2023). The antiviral activity of Ag‐NPs and ZnO‐NPs has also been reported for Tobacco Mosaic Virus (TMV) and Pepper Mild Mottle Virus (PMMoV) (Ahsan, 2020; Elbeshehy et al., 2022). The application of NPs promotes direct defence, reducing the dependency on excessive pesticide use and contributing to sustainable agricultural production (Asif et al., 2023). Moreover, NPs can also function as adjuvants or surfactants for chemical and biological pesticides, serving as nanocarriers for RNAs (interfering or double‐stranded) and exerting a synergistic effect with metabolites derived from *Bacillus thuringiensis* (Bt), which are widely used in pest control (Yin et al., 2023). Currently, several bioformulations are being evaluated, aiming to ensure that a smaller amount of applied product provides slower and more efficient target molecule delivery, supported by improved nutrient and water absorption (Francis et al., 2024c), ensuring that plants have the necessary resources to withstand prolonged and recurring stress periods (Dutta et al., 2022; Szopa et al., 2022).

Metal‐ and metal oxide‐derived NPs possess unique physicochemical properties that allow them to interact with plants and pathogens at the molecular level (Yiblet and Sisay, 2024). These interactions can strengthen plants' natural defences, making them more resistant to attacks from pests, diseases, and herbivores (Yadav et al., 2024). However, the application of NPs in agriculture still raises questions about their environmental sustainability and efficacy (Shukla et al., 2024; Vaidya et al., 2024). This mini‐review article explores how NPs can be formulated, applied, and effectively harnessed to mitigate biotic stress in crops while also considering their role in the broader context of climate change and food security.

## 2 NANOPARTICLE FORMULATIONS AND THEIR PROPERTIES

**FIGURE 1:**
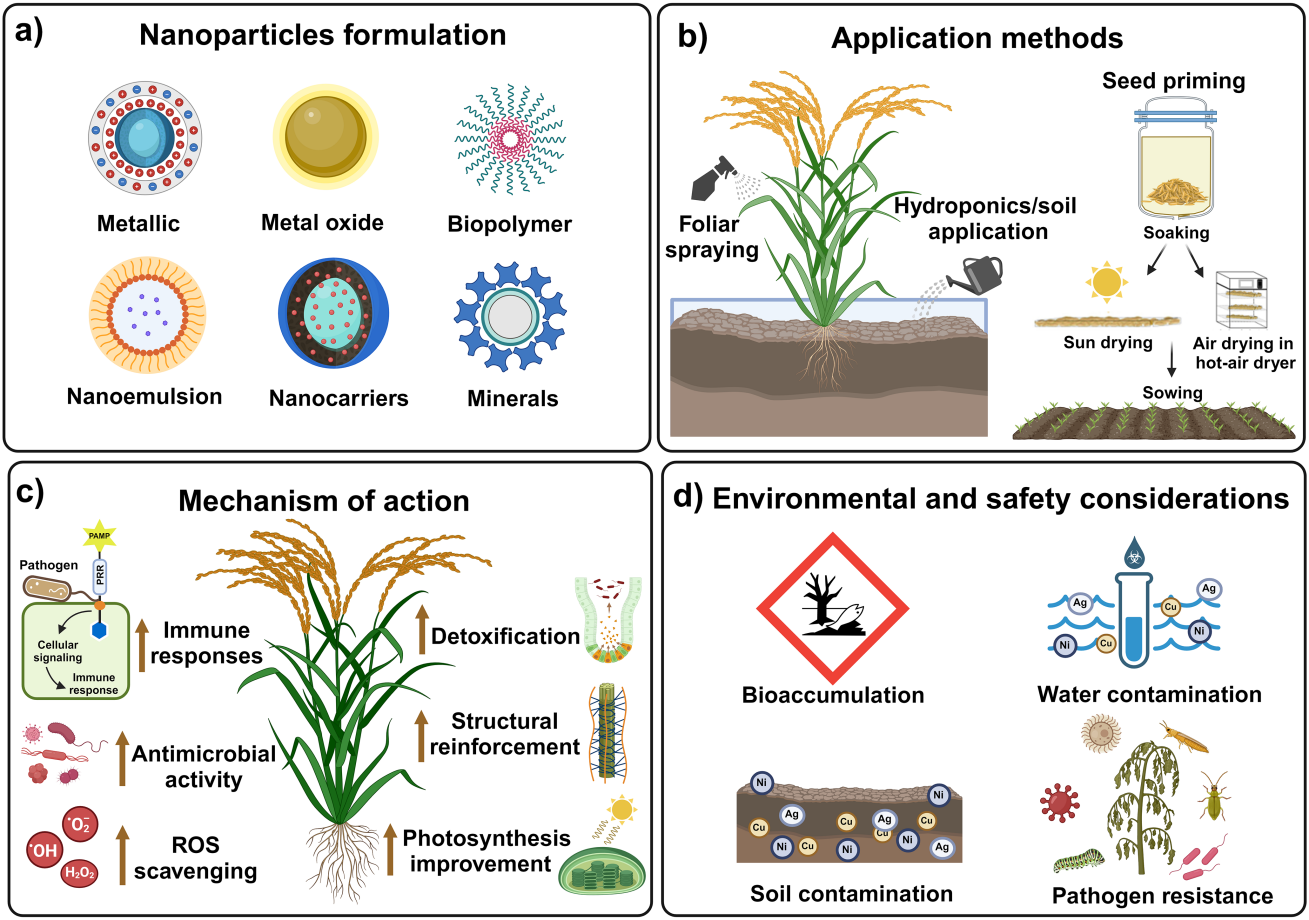
Nanoparticles (NP) formulation (a), application methods (b), mechanism of action (c), and environmental/safety considerations (d). ROS: Reactive Oxygen Species.

NPs used in agriculture can be classified into several categories based on their chemical composition and properties (Figure 1a). The most employed NPs for enhancing plant resistance to biotic stress include metal‐based NPs, such as silver (Ag‐NPs), zinc oxide (ZnO‐NPs), copper (Cu‐NPs), silica (SiO₂‐NPs), and selenium (Se‐NPs), as well as organic NPs like chitosan‐based ones (Alhebsi et al., 2024; Yiblet and Sisay, 2024). Each formulation offers specific advantages depending on its mode of action and the type of biotic stress it targets.

### 2.1 Silver NPs (Ag‐NPs)

Silver is known for its potent antimicrobial activity, which makes Ag‐NPs highly effective against a wide range of plant pathogens, including bacteria and fungi (Abasi et al., 2022). The antimicrobial action of Ag‐NPs is primarily due to their ability to release silver ions (Ag^+^), which disrupt the cell membranes of pathogens, damage the DNA, inhibit vital enzymes, and generate reactive oxygen species (ROS) that induce oxidative stress in the pathogens (Rodrigues et al., 2024). Smaller AgNPs (<20 nm) exhibit increased antimicrobial activity due to their higher surface area‐to‐volume ratio, which facilitates greater ion release and interaction with pathogens.

### 2.2 Zinc Oxide NPs (ZnO‐NPs)

ZnO‐NPs exhibit both antimicrobial properties and plant growth‐promoting effects (Verma et al., 2024b). Their small size and large surface area enable them to interact effectively with microbial cells, causing cell wall disruption and ROS generation (Yiblet and Sisay, 2024). ZnO‐NPs also enhance nutrient uptake in plants, contributing to improved plant vigour and resilience against pests and pathogens (Lv et al., 2022). ZnO formulations may be enhanced by doping with other metals (e.g. Copper) to increase their efficacy and broaden their spectrum of activity (Tryfon et al., 2022).

### 2.3 Selenium NPs (Se‐NPs)

Selenium, a micronutrient, plays a dual role as a nutrient and antioxidant (Lanza and Reis, 2021). Se‐NPs are particularly useful in mitigating oxidative stress induced by biotic factors, such as herbivory and pathogen attacks, scavenging ROS, protecting plant cells from oxidative damage and enhancing the production of defence‐related metabolites (Garza‐García et al., 2022). Se‐NPs have been shown to improve resistance to both biotic and abiotic stress factors, making them a versatile tool in sustainable agriculture (Samynathan et al., 2023). The effectiveness of Se‐NPs is influenced by their size, with smaller NPs having increased bioavailability and activity, making them more efficient in combating stress.

### 2.4 Silica NPs (SiO₂‐NPs)

SiO₂‐NPs are primarily used to enhance plant structural defences. Silica fortifies cell walls, making it more difficult for pests such as insects and mites to penetrate the plant tissues (Balakrishnan et al., 2024). Additionally, SiO₂‐NPs can trigger the activation of defence signalling pathways, such as those mediated by salicylic acid (SA) and jasmonic acid (JA), which are crucial for the plant's immune response (Naidu et al., 2023; Saw et al., 2023). The morphology of SiO₂‐NPs also plays a significant role in their ability to integrate into plant tissues and reinforce these structural defences.

### 2.5 Chitosan‐based NPs

Chitosan, a natural biopolymer derived from chitin, is often used in NP formulations due to its biodegradability and biocompatibility (Zhang et al., 2022), besides allowing controlled release of the active agent over time (Khezerlou et al., 2021). Chitosan NPs have been shown to induce systemic acquired resistance (SAR) in plants by triggering the expression of defence genes (Abd‐Ellatif et al., 2022). They also possess antifungal and antibacterial properties, making them effective in protecting plants against a broad range of pathogens (Kumar et al., 2021; Poznanski et al., 2023). Surface functionalization, such as chitosan coating, enhances the stability and controlled release of these NPs, extending their protective effects over a longer period.

## 3 APPLICATION METHODS OF NANOPARTICLES

The effectiveness of NPs in enhancing plant resistance to biotic stress depends not only on their formulation but also on how they are applied to crops (Figure 1b). The most common application methods include:

### 3.1 Foliar spraying

NPs are typically applied as foliar spray, where they are absorbed through the stomata or cuticle of the plant. This method ensures direct contact with the pathogens and provides a rapid response to biotic stress (Lv et al., 2022). Foliar sprays of Ag‐NPs and ZnO‐NPs have been shown to effectively control fungal and bacterial infections in crops like tomatoes, with smaller particles exhibiting better absorption efficiency due to their high surface area‐to‐volume ratio, which enhances their antimicrobial efficacy (Bouqellah et al., 2024; Hussain et al., 2024; Khatoon et al., 2024). Foliar spraying is widely used for its rapid response, though absorption efficiency can vary based on leaf characteristics and environmental conditions, with smaller particles generally showing higher absorption rates (Lv et al., 2022).

### 3.2 Soil/hydroponics application

Some NPs, such as Se‐NPs and SiO₂‐NPs, are applied to the soil or hydroponic solution to enhance root uptake and improve plant resistance from the ground up (Samynathan et al., 2023; Taha et al., 2023). Soil application is especially useful for NPs that improve nutrient uptake or fortify plant structures, such as silica, which strengthens plant cell walls, and Se‐NPs, which mitigate oxidative stress through root absorption. The particle size plays a critical role in improving root interaction and enhancing these effects (Bhat et al., 2021; Naidu et al., 2023). Soil and hydroponic applications provide longer‐term benefits by integrating NPs like SiO₂‐NPs and Se‐NPs into plant defence and structural pathways.

### 3.3 Seed priming

Seed priming with NPs involves soaking seeds in a solution containing NPs before planting (Francis et al. 2024c). This method can enhance seed germination, increase seedling vigour, and improve early resistance to pathogens and pests (Yang et al., 2024a). Seed priming with ZnO‐NPs, for example, has been shown to promote stronger seedlings with enhanced resistance to fungal infections. The small size of ZnO‐NPs aids in better absorption, which activates antioxidant defences and improves pathogen resistance (Farhana et al., 2022; Donia and Carbone, 2023). Seed priming strengthens seedlings from germination onward, with NP size and surface functionalization further enhancing antioxidant defences and resilience to biotic stress (Donia and Carbone, 2023).

## 4 MECHANISMS OF ACTION INSIDE PLANTS

Once NPs are applied, their mode of action within the plant varies depending on their composition and the specific biotic stress they are designed to mitigate (Figure 1c). Generally, NPs interact with plants through the following mechanisms:

### 4.1 Activation of plant immune responses

Certain NPs, like ZnO‐NPs and SiO₂‐NPs, activate plant immune signalling pathways that lead to SAR and allow plants to respond more rapidly to pathogen challenges (El‐Shetehy et al., 2021; Sofy et al., 2021; Du et al., 2022). These NPs can stimulate the production of defence‐related hormones, such as SA and JA, which enhance the plant's ability to fend off subsequent attacks from pathogens or herbivores (Vankova et al., 2017; Fujikawa et al., 2021; Dong et al., 2023).

### 4.2 Antimicrobial activity

Metal‐based NPs, such as Ag‐NPs and Cu‐NPs, have strong antimicrobial effects (Jali et al., 2024). Inside the plant, they disrupt pathogen cell walls, interfere with DNA replication, and generate ROS that damage the cell walls and DNA of pathogens, killing or at least inhibiting the growth of fungi, bacteria, and other microbial invaders (Sahoo et al., 2023; Kaleem et al., 2024). This antimicrobial action can be systemic, meaning that even parts of the plant not directly exposed to the NPs benefit from increased resistance (Wang et al., 2019).

### 4.3 ROS scavenging and detoxification

Biotic stressors, such as insect herbivory or pathogen invasion, often lead to the overproduction of ROS in plants, causing oxidative damage (Tyagi et al., 2022). Se‐NPs, in particular, act as antioxidants by neutralizing ROS, protecting plant tissues from oxidative stress, and improving overall plant resilience (Basit et al., 2023).

### 4.4 Structural reinforcement

NPs such as SiO₂‐NPs integrate into the plant's cell walls, making them physically stronger and more resistant to penetration by herbivores or pathogens (El‐Shetehy et al., 2021). This structural fortification adds a physical barrier to the plant's defence strategy, making it harder for pests like insects and mites to feed on the plant tissues (Balakrishnan et al., 2024).

## 5 STRATEGIES USING NANOPARTICLES TO INCREASE PLANT RESISTANCE TO BIOTIC STRESSES

Different formulations of NPs have been tested in the last few years, with the aim of increasing plant resistance to stressful biotic conditions, such as fungi/bacterial infection and herbivore infestation. To have a glimpse of what is on the cutting edge of knowledge, we present in Table 1 the most recent approaches (published in 2024) that used NPs to enhance plant protection against pests and pathogens. We identified 15 studies: six focused on fungi, seven on bacteria, and only two on herbivores. All experiments were conducted under greenhouse conditions, indicating that research in this area is still in its early stages and that field trials are needed to assess the real effectiveness of these nanotechnologies. Most studies (11 out of 15) applied NPs via foliar spraying.

**TABLE 1:** Strategies using nanoparticles to increase plant resistance to biotic stresses. All experiments were conducted under greenhouse conditions.

Strategies using nanoparticles to increase plant resistance to fungal infections						
Crop	Nanoparticle type	Application method and amount applied	Target organism	Stress resistance outcome	Other physiological traits	Reference
*Oryza sativa* L. (cv. Xiang Liang 900)	Moringa chitosan nanoparticles (M‐Cs‐NPs)	Foliar spraying ‐ 200 mg L^‐1^	*Magnaporthe oryzae* strain 70‐15, the causal agent of rice blast disease	Inhibition (78%) of pathogen growth, integrity, and colonization	Enhanced antioxidant enzyme activity and plant health / Improved photosynthesis, gas exchange, and nutritional profile of diseased rice plants / Upregulation of defense‐related genes	Hafeez et al. (2024)
*Oryza sativa* L. (cv. BRRI Dhan48)	Ag‐NPs	Foliar spraying ‐1.5, 3.0, 4.5, 6.0, 7.5, 15.0, 22.5, 30.0, and 37.5 ppm (two applications, two and ten days after pathogen inoculation)	*Rhizoctonia solani*, the causal agent of sheath blight disease	Inhibition of mycelial growth (mostly with 7.5 and 37.5 ppm)	‐	Islam et al. (2024)
*Oryza sativa* L. (cv. Nanjing 9108)	Pectin‐coated iron metal‐organic framework nanoparticles (Fe‐MOF‐PT‐NPs)	Foliar spraying ‐ 200, 400, and 800 mg L^‐1^	*Rhizoctonia solani*, the causal agent of sheath blight disease	Inhibited mycelial growth and sclerotia production / Increased fungal DNA damage / Inhibition of fungal nucleotide metabolism	Reduced oxidative damage / Increased photosynthesis, jasmonate signaling, and expression of disease resistance genes	Yang et al. (2024b)
*Triticum aestivum* L. (unknown cv.)	ZnO‐NPs	Ears spraying ‐ 15 μg mL^‐1^	*Fusarium graminearium* strain PH‐1 (NRRL 31084)	Inhibition of fungal growth, colony formation, spore germination, and mycotoxin (deoxynivalenol) production / Increased fungal cell wall damage and ROS generation	Increased activity of antioxidant enzymes, plant growth and seed yield	Ibrahim et al. (2024)
*Solanum lycopersicum* L. (cv. Kashi Amrit)	Ag‐NPs	Foliar spraying ‐ 20 ppm	*Alternaria solani* (MTCC N^o^ 2101), the causal agent of early blight disease	Enhanced plant resistance by reducing stress parameters and stress enzyme activity	Increased levels of antioxidants and chlorophyll	Narware et al. (2024)
*Cucumis sativus* L. (cv. Super Dominus)	Celery seed essential oil (CSEO)‐loaded chitosan nanoparticles (CSEO‐LC‐NPs)	Foliar spraying ‐ 50, 100, 200, and 400 μg mL^‐1^	*Podosphaera fusca* strain HS101, the causal agent of cucumber powdery mildew	Reduced disease severity of powdery mildew	Increased phenolic and flavonoid compounds, chlorophyll concentration, enzyme activities, and gene transcriptions	Soleimani et al. (2024)

Strategies using nanoparticles to increase plant resistance to bacterial infections						
Crop	Nanoparticle type	Application method and amount applied	Target organism	Stress resistance outcome	Other physiological traits	Reference
*Oryza sativa* L. (cv. II You 023)	NiO‐NPs	Foliar spraying ‐ 200 mg mL^‐1^	*Xanthomonas oryzae* pv. *oryzae*, the causal agent of bacterial leaf blight	Reduction in diseased leaf area (up to 74%)	Improved plant growth and biomass	Abdallah et al. (2024)
*Oryza sativa* L. (cv. II You 023)	CuO‐NPs	Foliar spraying ‐ 200 mg mL^‐1^	*Xanthomonas oryzae pv. oryzae* (strain GZ 0003), the causal agent of bacterial leaf blight	Reduced symptoms of bacterial leaf blight	Increased plant growth and biomass	Ogunyemi et al. (2024)
*Arachis hypogaea* L. (cv. Zhonghua12)	SiO_2_‐NPs	Hydroponics ‐ 100 and 500 mg L^‐1^	*Ralstonia solanacearum*, the causal agent of bacterial wilt disease	Decreased disease indices (up to 55%)	Increased plant growth and activity of antioxidant enzymes / Reduced levels of oxidative stress / Enhanced salicylic acid (SA) contents by regulating the expression of SA‐metabolism genes	Deng et al. (2024)
*Daucus carota* L. (cv. Rose Red)	MnO_2_‐NPs	Foliar spraying ‐ 0.10 g L^‐1^	*Ralstonia solancearum* (bacterial wilt pathogen) and *Meloidogyne incognita* (root‐knot nematode), the causal agents of wilt disease complex	Reduced wilt disease indices / Reduced number of galls per root system / Reduced nematode population	Increased plant growth and chlorophyll/carotenoid/proline contents	Siddiqui et al. (2024)
*Solanum lycopersicum* L. (cv. Campbell 33)	Chitosan embedded with ZnO nanoparticles and hydroxyapatite (ZnO‐NP‐Hap@Cs)	Foliar spraying ‐ 200 μg mL^‐1^ (two applications with a 4‐day interval)	*Pseudomonas syringae* pv. *tomato* (strain DC3000), the causal agent of bacterial speck disease	Reduced disease severity of bacterial speck	‐	Esserti et al. (2024)
*Solanum lycopersicum* L. (cvs. Ujjawal and Xin‐Feng)	Ag‐NPs	Foliar spraying ‐ 10, 20, 30, and 40 ppm	*Clavibacter michiganensis* ssp. *michiganensis*, the causal agent of bacterial canker disease	Reduced bacterial canker disease incidence	Increased plant growth and chlorophyll/proline/total soluble sugar/relative water contents / Increased membrane stability index and catalase activity	Hussain et al. (2024)
*Solanum lycopersicum* L. (unknown cv.)	Si‐NPs	Soil application ‐ 30, 60, 90, 120, and 150 ppm	*Ralstonia solancearum* (phylotype 1, biovar 3), the causal agent of bacterial wilt disease	Reduced pathogen population in the soil and disease index in tomato plants	Increased plant growth and photosynthesis / Increased activity of antioxidant enzymes / Increased expression of resistance genes / Increased non‐enzymatic antioxidant capacity / Reduced levels of oxidative stress	Wei et al. (2024)

Strategies using nanoparticles to increase plant resistance to herbivore infestation						
Crop	Nanoparticle type	Application method and amount applied	Target organism	Stress resistance outcome	Other physiological traits	Reference
*Vigna radiata* L. (cv. DGGV‐2)	Ag‐NPs and Cu‐NPs	Seed priming + foliar spraying ‐ 1,000, 1,500, and 2,000 ppm	*Callosobruchus chinensis* L. (pulse beetle)	Strong insecticidal effect (100% mortality of bruchids) / Reduction in post‐harvest losses	Improved seed quality by enhancing catalase and a‐amylase activities in storage seeds / Increased plant growth, seed yield, and seed quality	Hemalatha et al. (2024)
*Capsicum annuum* L. (cvs. Arka Sweta, Arka Meghana, and Arka Haritha)	Chitosan (CS), Chitosan‐Silver (CSAg), and Chitosan‐Copper (CSCu) nanoparticles (CS‐NPs, CSAg‐NPs, CSCu‐NPs)	Foliar spraying ‐ 20, 40, 60, 80, and 100 ppm	Thrips (unknown species)	Protection against thrips by 70‐85%	Enhanced contents of chlorophyll (up to 75%), carotenoids (up to 30%), total phenolics (up to 45%), total flavonoids (up to 125%), reducing sugars (up to 40%), total antioxidant activity (up to 82%), FRAP (up to 100%), and total capsaicinoids (up to 142%) / Enhanced antioxidant capacity (FRAP and DPPH)	Mawale and Giridhar (2024)

Among the six strategies using NPs to boost plant resistance to fungal infections, three targeted rice plants infected by *Magnaporthe oryzae*, the causal agent of rice blast disease (Hafeez et al., 2024), and *Rhizoctonia solani*, the causal agent of sheath blight disease (Islam et al., 2024; Yang et al., 2024b). The remaining three studies focused on wheat plants infected by *Fusarium graminearum* (Ibrahim et al., 2024), tomato plants infected by *Alternaria solani*, the causal agent of early blight disease (Narware et al., 2024), and cucumber plants infected by *Podosphaera fusca*, the causal agent of cucumber powdery mildew (Soleimani et al., 2024). All six studies reported enhanced plant resistance, evidenced by reduced fungal growth, integrity, and colonization, as well as lower disease severity. Silver nanoparticles (Ag‐NPs) applied to rice and tomatoes exhibited strong antimicrobial effects through ROS generation, leading to the pathogen cell wall and DNA disruption and the upregulation of defence genes, including those involved in JA signalling (Hafeez et al., 2024; Islam et al., 2024; Narware et al., 2024). Additional protective mechanisms included strengthened antioxidant systems and increased levels of phenolic and flavonoid compounds. Defence‐related and disease‐resistance genes were upregulated, contributing to improved photosynthesis, plant health, growth, and yield. SiO₂‐NPs were also found to reinforce plant cell walls, enhancing physical protection and activating stress response pathways (Soleimani et al., 2024). Silver nanoparticles (Ag‐NPs) were the only NP used in multiple strategies at concentrations of 1.5‐37.5 ppm for rice (Islam et al., 2024) and 20 ppm for tomatoes (Narware et al., 2024).

Among the seven strategies that utilized NPs to enhance plant resistance to bacterial infections, three focused on tomato plants infected by *Pseudomonas syringae*, the causal agent of bacterial speck disease (Esserti et al., 2024), *Clavibacter michiganensis*, the causal agent of bacterial canker disease (Hussain et al., 2024), and *Ralstonia solanacearum*, the causal agent of bacterial wilt disease (Wei et al., 2024). Two strategies targeted rice plants infected by *Xanthomonas oryzae*, the causal agent of bacterial leaf blight (Abdallah et al., 2024; Ogunyemi et al., 2024). The remaining two studies investigated peanuts and carrots infected by *Ralstonia solanacearum*, the causal agent of bacterial wilt disease (Deng et al., 2024; Siddiqui et al., 2024). In tomato plants, Ag‐NPs and Cu‐NPs induced a significant increase in SA metabolism, crucial for enhancing SAR and boosting plant immune responses (Esserti et al., 2024). All seven studies reported reductions in diseased leaf area and symptoms, accompanied by increases in antioxidant capacity, membrane stability, and levels of chlorophyll, carotenoids, proline, and total soluble sugars. Genes associated with resistance and SA metabolism were upregulated, resulting in enhanced SA content and improved plant growth and biomass. Most strategies (five out of seven) employed metal oxide NPs, including NiO‐NPs, CuO‐NPs, SiO_2_‐NPs, MnO_2_‐NPs, and ZnOHap@Cs‐NPs, while the others used Ag‐NPs (Hussain et al., 2024) and Si‐NPs (Wei et al., 2024). SiO₂‐NPs, applied in both bacterial and fungal treatments, not only strengthened plant structures but also triggered stress response pathways, further contributing to pathogen resistance (Siddiqui et al., 2024; Wei et al., 2024).

The two strategies that utilized NPs to enhance plant resistance to herbivore infestation focused on mung bean infested by *Callosobruchus chinensis* (Coleoptera: Bruchidae) (Hemalatha et al., 2024) and pepper infested by thrips (Thysanoptera) (Mawale and Giridhar, 2024). Seed priming and foliar spraying of Ag‐NPs and Cu‐NPs (1,000‐2,000 ppm) showed a strong insecticidal effect against mung bean bruchids, improving plant growth, seed yield and quality, and reducing post‐harvest losses (Hemalatha et al., 2024). In peppers, foliar spraying (20‐100 ppm) of different chitosan‐based NPs (CS‐NPs, CSAg‐NPs, and CSCu‐NPs) provided up to 85% protection against thrips, which was associated with increased production of chlorophyll, carotenoids, phenolics, flavonoids, reducing sugars, antioxidant compounds, and capsaicinoids (Mawale and Giridhar, 2024). The ability of chitosan‐based NPs to induce the synthesis of secondary metabolites highlights their role in activating plant defence mechanisms against herbivory and pathogen infection.

The recent advancements in NP‐based strategies for enhancing plant resistance to biotic stresses highlight not only the efficacy of NPs but also the diverse mechanisms by which they operate within plants. These findings illustrate the complex interactions between NPs and plant defence systems, underscoring the importance of understanding the specific NP properties and application methods to maximize their protective effects against various biotic stresses.

## 6 CLIMATE CHANGE AND THE ROLE OF NANOPARTICLES IN SUSTAINABLE AGRICULTURE

Climate change is predicted to worsen biotic stress by altering the dynamics of pest populations and pathogen spread (Proesmans et al., 2021). Warmer temperatures, for example, can accelerate the life cycles of insects and facilitate the spread of plant diseases. In regions where water scarcity is becoming a critical issue, plants may also face combined stress from both abiotic (drought, heat) and biotic factors (Shelake et al., 2024). NPs offer a sustainable solution to these challenges, as they can reduce the need for chemical pesticides and fertilizers, which are environmentally damaging and contribute to greenhouse gas emissions (Vaidya et al., 2024; Wasule et al., 2024).

Nanotechnology enables precise targeting for the delivery of molecules in the control of pests and pathogens, reducing collateral damage to beneficial organisms such as pollinators and soil microbiota (Zhang et al., 2021; Lowry et al., 2024). Furthermore, by enhancing plant resilience to both biotic and abiotic stressors, NPs can help improve crop yields even under suboptimal growing conditions caused by climate change (Fernández‐Triana et al., 2024). For example, the application of Se‐NPs has been shown to enhance drought tolerance in addition to its role in mitigating pathogen attacks, making it a valuable tool in climates where water availability is uncertain (El‐Saadony et al., 2021).

In the context of climate change, nanoformulations like chitosan‐salicylic acid (Ch‐SA‐NP) and zinc sulfate (Zn‐NP) may become increasingly valuable tools for managing pest and pathogen pressures in agriculture. For example, Ch‐SA‐NP and Zn‐NP were shown to control the bacterium *Puccinia striiformis* f. sp. *tritici* (the causal agent of stripe rust) in two wheat genotypes by significantly enhancing defence‐related enzyme activities, including phenylalanine ammonia lyase, tyrosine ammonia lyase, and polyphenol oxidase (Hembade et al., 2022). They also triggered pathogenesis‐related (PR) gene expression and increased sugar accumulation, contributing to pathogen resistance. Such effects are particularly relevant as climate change is predicted to alter the dynamics of pest populations and pathogen spread, potentially increasing the incidence and severity of diseases. Furthermore, chitosan‐based nanocapsules with eugenol (90 μg mL^‐1^) activated defence genes (*Chitinase* and *β‐1,3‐glucanase*) in potato plants and suppressed virulence genes (*PhcA*, *XpsR*, and *HrpG*) of *Ralstonia solanacearum*, a major pathogen responsible for bacterial wilt in solanaceous crops (Oluoch et al., 2022). Given the potential for climate change to exacerbate pathogen virulence and spread, these nanoformulations offer a sustainable alternative to conventional pesticides, which often have environmentally damaging effects. Through targeted activation of plant immune responses, these NPs present a more ecologically responsible strategy for managing agricultural disease in a warming world.

## 7 ENVIRONMENTAL AND SAFETY CONSIDERATIONS

Despite the many advantages of using NPs in agriculture, there are valid concerns about their long‐term environmental impact (Figure 1d). NPs, particularly metal‐based ones, may accumulate in the soil and water, potentially affecting non‐target organisms or disrupting microbial communities (Bordin et al., 2024). For instance, studies indicate that Cu‐NPs can accumulate in plant tissues, leading to potential toxicity and environmental disruptions. Cucumber plants treated with Cu‐NPs accumulated high levels of Cu ions in the roots, causing phytotoxicity, oxidative stress, membrane damage, and the overexpression of Cu‐Zn‐SOD enzyme genes (Mosa et al., 2018). Despite the desired fungicidal and bactericidal effects, other studies highlight the risks of bioaccumulation and toxicity associated with metal NPs (Li et al., 2017). Moreover, the overuse of antimicrobial NPs, such as Ag‐NPs and ZnO‐NPs, could promote the development of resistant microbial strains, further complicating their environmental safety (Kamat and Kumari, 2023). Given this conflicting data, we recommend that the application of metallic and metal oxide NPs be conducted with caution, and further field studies should be carried out to assess the safety and toxic effects of these nanomaterials.

Overuse of certain NPs could also lead to the development of resistance in pathogens, similar to how the overuse of antibiotics can lead to resistant bacterial strains (Kamat and Kumari, 2023). To address these concerns, researchers are exploring the development of green synthesis methods for NPs, which involve using plant extracts or other natural materials to produce environmentally friendly NPs (Peta and Singh, 2024). These “green” NPs are not only biodegradable but also tend to have fewer toxic effects on non‐target organisms (Wasule et al., 2024). Additionally, integrating NP applications with sustainable agricultural practices, such as integrated pest management (IPM), can help mitigate potential risks while maintaining their protective benefits (Zain et al., 2024). However, further field studies are necessary to assess the true long‐term ecological consequences of NP applications.

## 8 PERSONAL INSIGHTS

The use of NPs to enhance plant resistance to biotic stresses represents an emerging strategy in sustainable agriculture, particularly as climate change intensifies crop vulnerability to pests and pathogens. Traditional disease management relies heavily on chemical pesticides, which can lead to environmental contamination and resistance development in target organisms. In contrast, NPs offer a more targeted and efficient alternative, capable of delivering antimicrobial compounds precisely where they are needed. Certain NPs have also been reported to stimulate plant immune responses, potentially reducing disease severity through primed defence mechanisms. Additionally, nanoscale materials can improve nutrient availability and act as carriers for bioactive molecules, strengthening plant health and enhancing stress resilience.

As climate conditions become increasingly unpredictable, adaptable disease management approaches are essential. NPs can be tailored to suit specific crops and environments, offering a customizable strategy that balances plant protection with ecological sustainability. Some studies suggest that NPs can suppress pathogen activity without negatively affecting beneficial soil microbiota, an important consideration for long‐term agricultural health. Furthermore, beyond their role in pathogen control, NPs may support plant growth under stress by enhancing root architecture, nutrient uptake, and photosynthetic efficiency. However, despite their promise, NP‐based solutions require careful risk assessment, particularly regarding their long‐term interactions with the environment. Ensuring their safe and scalable application will be critical for integrating these technologies into mainstream agricultural practices.

While there is growing evidence that NPs can help reduce biotic stress, the exact mechanisms behind their effects are still not fully understood. One of the key questions that remain is whether NPs directly impact pests and pathogens through toxic or antimicrobial properties or if their benefits come from indirect effects, such as priming plant defences. For example, although we know that metals like silver (Ag‐NPs) and copper (Cu‐NPs) have antimicrobial activity, some studies suggest that NPs also influence plant signalling pathways, leading to changes in ROS, phytohormones, and defence metabolites, which in turn improve plant resistance. However, what is missing in current research is a direct comparison between how NPs affect the pest alone (NP‐pest) and how they interact with the pest‐plant system (NP‐pest‐plant). Understanding whether the benefits are due to direct toxicity to pests or indirect plant defence mechanisms is essential for refining NP‐based strategies for pest control. Additionally, important concerns around the long‐term ecological effects of NPs, such as bioaccumulation, potential toxicity to non‐target organisms, and impacts on soil microbiomes, have not been fully addressed. Future studies should combine molecular approaches, like transcriptomics and metabolomics, to better understand the mechanisms behind NP‐mediated plant resistance while also considering their safety, environmental impact, and practical use in agriculture. A collaborative, interdisciplinary approach is key to unlocking the full potential of NPs in sustainable crop protection.

## 9 CONCLUSION

The application of NPs in enhancing plant resistance to biotic stress presents a promising strategy for addressing the dual challenges of climate change and food security. Metal‐based NPs, such as Ag‐NPs and ZnO‐NPs, alongside organic formulations like chitosan‐based NPs, have demonstrated efficacy in boosting plant defences against a range of biotic stresses, including fungi, bacteria, and herbivores. These NPs can be applied through various methods, such as foliar spraying, soil treatments, and seed priming, offering precise and targeted protection. However, critical concerns remain regarding the long‐term environmental consequences of NP accumulation, bioaccumulation, and potential toxicity to non‐target organisms. Moreover, the sustainability and scalability of NP production methods must be addressed to ensure cost‐effectiveness and minimal ecological disruption. While the early‐stage research indicates substantial potential, more comprehensive field‐based studies and investigations into the ecological fate of NPs are essential to fully assess their environmental safety. By integrating NPs with sustainable practices like IPM, nanotechnology could contribute to the development of more resilient and environmentally conscious agricultural systems, helping meet the growing demand for food in a changing climate.

## AUTHOR CONTRIBUTIONS

All authors listed have made a substantial, direct, and intellectual contribution to the work and approved it for publication.

## CONFLICT OF INTEREST

The authors declare no potential conflict of interest.

## Data Availability

Data sharing is not applicable to this article as no new data were created or analyzed in this study.
